# YOLOFM: an improved fire and smoke object detection algorithm based on YOLOv5n

**DOI:** 10.1038/s41598-024-55232-0

**Published:** 2024-02-24

**Authors:** Xin Geng, Yixuan Su, Xianghong Cao, Huaizhou Li, Linggong Liu

**Affiliations:** College of Building Environment Engineering, Zhengzhou University of Light Industry, Zhengzhou, 450006 China

**Keywords:** Electrical and electronic engineering, Computer science, Information technology, Electrical and electronic engineering, Computer science, Information technology

## Abstract

To address the current difficulties in fire detection algorithms, including inadequate feature extraction, excessive computational complexity, limited deployment on devices with limited resources, missed detections, inaccurate detections, and low accuracy, we developed a highly accurate algorithm named YOLOFM. We utilized LabelImg software to manually label a dataset containing 18644 images, named FM-VOC Dataset18644. In addition, we constructed a FocalNext network, which utilized the FocalNextBlock module from the CFnet network. This improves the integration of multi-scale information and reduces model parameters. We also proposed QAHARep-FPN, an FPN network that integrates the structure of quantization awareness and hardware awareness. This design effectively reduces redundant calculations of the model. A brand-new compression decoupled head, named NADH, was also created to enhance the correlation between the decoupling head structure and the calculation logic of the loss function. Instead of using the CIoU loss for bounding box regression, we proposed a Focal-SIoU loss. This promotes the swift convergence of the network and enhances the precision of the regression. The experimental results showed that YOLOFM improved the baseline network’s accuracy, recall, F1, mAP50, and mAP50-95 by 3.1%, 3.9%, 3.0%, 2.2%, and 7.9%, respectively. It achieves an equilibrium that combines performance and speed, resulting in a more dependable and accurate solution for detection jobs.

Fires often cause serious casualties and property damage. Therefore, early detection and accurate identification of fires are crucial for reducing losses and protecting people’s lives and property. Traditional fire detection technologies rely primarily on temperature, light, and smoke sensors. However, these approaches have several limitations, such as limited detection range and low detection accuracy. The development of computer vision has resulted in substantial enhancements in tackling these challenges. According to Celik et al.’s YCbCr separation^1^, “they analyzed shape, color, and texture to identify fire smoke.” Yamagishi et al.^2^ employed color CCD cameras. Nevertheless, these approaches performed well only in properly lit and uncomplicated contexts but struggled in complex environments with insufficient lighting, resulting in poor detection and incorrect alerts. Support vector machine (SVM)^3^ are inadequate at detecting fires because they frequently generate false alarms when cameras move or function in vibrating surroundings. Chi et al.^4^ addressed several problems related to videos captured by stationary cameras. However, difficulties such as restricted location choices and expensive upkeep continue to exist. Toreyin et al.^5^ proposed a real-time video processing system but encountered performance degradation issues when dealing with high-resolution videos.

With the upgrading of computer hardware and the development of deep learning technology, an increasing number of deep learning algorithms are being utilized in fire detection. The algorithms currently used include Faster R-CNN^6^, YOLO^7^, EfficientDet^7^, YOLOX^8^, SSD^9^, RetinaNet^10^, and CenterNet^11^. Chaoxia et al.^6^ reduced Faster R-CNN false alarms by adopting a color-guided anchoring strategy. However, this improvement came at the expense of increased computational complexity. Xu et al.^7^ improved EfficientDet to detect forest fires. Nevertheless, acquiring complete global information remained a challenge. Liau et al.^9^ improved the detection speed of SSD networks, but accuracy in complicated circumstances still has to be improved. To boost network robustness, Li et al.^11^ proposed a lightweight backbone network and anchor-free detection methods. However, this improvement has serious drawbacks when dealing with complicated scenarios with shifting lighting conditions. These deep learning algorithms employed comprehensive analyses of various fire features, such as color, texture, and shape. In contrast to traditional visual processing algorithms, they show greater resilience in complex scenarios, decreasing the frequency of incorrect detection and better meeting the requirements of complex tasks. However, challenges remain, including increased computational complexity, data acquisition difficulties, and potential interference when handling high-resolution videos or large data streams.

YOLO, as a single-stage object detection algorithm, is widely utilized in fire detection because of its distinctive network structure and outstanding performance. Several modifications and optimizations have been proposed, including ELASTIC-YOLOv3^12^ for urban nighttime fire detection; YOLOv3^13^ for forest fire detection; and YOLOv4^14^ for fire detection at construction sites. Several recent versions, including YOLOv5^15^, YOLOv6^16^, YOLOv7^17,18^, and YOLOv8^19,20^, have been developed to address various fire detection tasks. Despite the potential of YOLOv7^17^ in ship fire detection, there are still difficulties to address in intricate fire scenarios. Luo et al.^21^ used Swin Transformer, CBAM, and Slim Neck to make it easier to identify lab fires, but this increased the computational load of the network too much. Zhang et al.^8^ proposed T-YOLOX to detect multiple targets but have accuracy issues in complex fire circumstances. The latest YOLO version is YOLOv8^19,20^. This version greatly improves upon its prior version. Nevertheless, due to network-related upgrades, the official version still exhibits some degree of instability. YOLOv5^15^ is the previous version of the YOLO series. Compared to YOLOv8, the network is significantly streamlined, resulting in a reduced model size and improved efficiency in deployment and operation. In addition, YOLOv5 utilizes a less computationally demanding backbone network and several optimization techniques to improve the efficiency of object detection, making it highly suitable for real-time scenarios. Furthermore, YOLOv5 provides a straightforward API interface and pre-trained model, making it a convenient and user-friendly choice. The YOLOv5n version demonstrates outstanding efficiency in resource-constrained contexts. However, it shows shortcomings in fire detection capabilities when compared to larger YOLO versions.

Because object detection algorithms have been used successfully in fire detection, we used images of fire and smoke to improve YOLOv5n and proposed YOLOFM, an effective fire and smoke object detection algorithm that can quickly and accurately detect different fire scenes. The main contributions of this paper can be summarized as follows:

- To address the limited availability and inadequate quality of publicly accessible fire object detection datasets, we created a dataset named FM-VOC Dataset18644. The dataset contains 16,844 images depicting fire and smoke. In addition, we employed image enhancement methods such as flipping, rotating, and adjusting image brightness to preprocess the dataset, which improved the quality and quantity of data for the experiments.
- Considering the importance of YOLOv5n’s fusion network in multiscale feature fusion and the issue of insufficient feature fusion caused by limited parameters, we proposed the FocalNext network. This network takes inspiration from the design concept of the CFNet network^22,23^ and incorporates the FocalNextBlock focusing module to reconstruct the backbone network. This network can integrate feature fusion operations into the backbone network, simultaneously merging detailed local features and broad global characteristics. This allows the fusion network to function efficiently in the subsequent phase.
- We integrated network quantization and reparameterization methods to construct a QARepVGG-style^24,25^ feature pyramid network QAHARep-FPN. It solves the issue of detection accuracy loss during network quantization and re-parameterization, as well as the difficulty of completing complex fire and smoke detection tasks on mobile devices and embedded systems with constrained hardware resources. This design achieves an effective balance between detection accuracy and inference speed.
- The original YOLOv5n head network uses an integration and sharing method for classification and regression tasks. However, this method results in inadequate focus on the bounding box regression task and uneven feature acquisition. To address this issue, we proposed a new asymmetric decoupled head (NADH) that uses multi-level channel compression technology to address the issue of insufficient feature learning in bounding box regression tasks^26,27^.
- The original YOLOv5n’s CIoU Loss has obvious flaws in effectively balancing the weights of positive and negative samples, handling overlapping targets, and addressing the relative ratio of length and width between detection and prediction boxes. To address these concerns, we proposed a new loss function called Focal-SIoU Loss. This loss function combines SIoU Loss^28^ and Focal L1 Loss^29–37^. More loss-related parameters, such as angle, distance, form, and IoU, are considered. This effectively increases the model’s convergence speed during training and improves the accuracy of the bounding box regression.

## YOLOFM network

**Figure 1:**
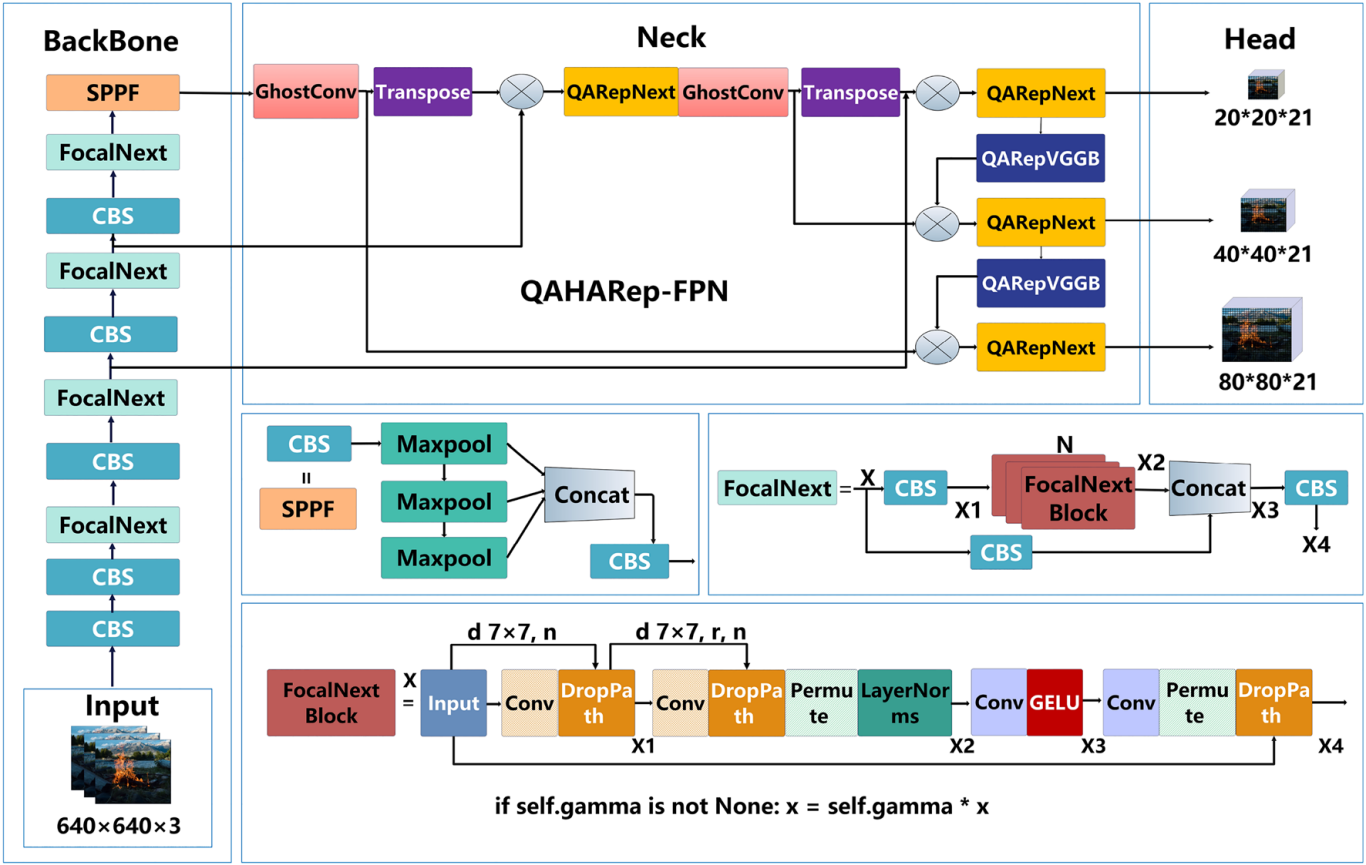
The YOLOFM network structure.

The YOLOFM network architecture consists of four parts: the input, backbone, neck, and head. The network structure of YOLOFM is shown in Fig. 1. When conducting fire and smoke detection, begin by resizing the image in the input network and standardizing the pixel values to a range of 0 to 1. The shape of the input image after preprocessing in this paper is (640, 640, 3). Subsequently, the preprocessed images are conveyed to the YOLOFM backbone network. Initially, perform two conventional convolution operations on the features with shape (640, 640, 3), yielding features with dimensions (320, 320, 12) and (320, 320, 64). Subsequently, three FocalNext+CBS feature extraction processes are executed, yielding features with dimensions of (160, 160, 128), (80, 80, 256), and (40, 40, 512), respectively. Next, execute the FocalNext+SPPF operation to acquire a feature with dimensions of (20, 20, 1024). During the feature extraction phase, YOLOFM generates numerous feature layers to detect objects, resulting in a total of three feature layers. The three feature layers are situated at distinct places inside the backbone network, namely the middle layer, the middle-lower layer, and the bottom layer. The dimensions of the three feature layers are as follows: feature1 = (80, 80, 256), feature2 = (40, 40, 512), and feature3 = (20, 20, 1024). The FPN network is formed in the neck network after acquiring three feature layers that are proven to be useful. The building approach involves performing a $1\times 1$ GhostConv convolution on the feature layer of feature3, which has dimensions (20, 20, 1024), to change the channel and generate feature5. Feature5 utilizes transpose operations to perform upsampling and then merges the result with feature2, which has dimensions of (40, 40, 512). Next, it employs QARepNeXt to extract features, resulting in the feature layer feature5_Transpose with dimensions of (40, 40, 512). The feature5_Transpose=(40, 40, 512) does a $1\times 1$ GhostConv convolution to modify the channel and obtain feature4. Feature4 is subsequently upsampled and merged with feature1=(80,80,256) using the Transpose operation. Afterward, QARepNeXt is utilized to extract features, which leads to the creation of the feature layer feature3_out=(80, 80, 256). The feature3_out=(80, 80, 256) uses a $3\times 3$ QARepVGGB convolution for downsampling, followed by the merging of the downsampled feature3 with feature4. QARepNeXt is subsequently employed for feature extraction, getting the feature layer feature2_out with dimensions (40, 40, 512). The feature2_out=(40, 40, 512) performs a $3\times 3$ QARepVGG convolution to reduce the size and then combines it with feature5 after downsampling. QARepNeXt is subsequently employed for feature extraction, getting the feature layer feature1_out with dimensions of (20, 20, 1024). The FPN yields three important features: (20, 20, 1024), (40, 40, 512), and (80, 80, 256). Subsequently, we employ these three features as input for the YOLOFM Head network to acquire prediction outcomes. Given that our categories are restricted to “fire” and “smoke”, the resulting forms of the three feature layers are ultimately (20, 20, 21), (40, 40, 21), and (80, 80, 21).

### Proposed FocalNext network

Traditional YOLO models employ the backbone network to extract multiscale features, which are subsequently fused in lightweight networks such as the feature pyramid network (FPN). However, the lightweight YOLOv5n model has fewer parameters assigned for the FPN network compared to the backbone network. We proposed a FocalNext network, which incorporates the FocalNextBlock focusing module and draws inspiration from the architecture of CFNet^22,23^ to improve the integration of features without compromising the lightweight design. This network can integrate feature fusion operations into the backbone network, simultaneously merging detailed local features and broad global characteristics. This increases the number of parameters that can be used for feature fusion while still allowing the model to benefit from the weights obtained from pre-training.

The structure of the FocalNext network is shown in Fig. 1. The structure consists of a skip connection and a series of stacked FocalNextBlock modules. The input tensor *X* processes a sub-path and an independent convolution operation before combining it with the feature $X_2$ that has undergone FocalNextBlock stacking to produce $X_3$. Finally, the combined feature $X_3$ undergoes a convolutional operation to produce the ultimate output $X_4$. For feature fusion and multilayer processing, the FocalNext network used skip connections to make the network better at showing details. This approach effectively mitigates the issue of gradient disappearance that arises with increasing network depth.

The FocalNextBlock is a focusing block within the FocalNext network. The module combines two skip connections and extended depth convolution, which lets fine-grained local interactions and coarse-grained global interactions merge at the same time. Fig. 1 illustrates the internal structure of the FocalNextBlock. The first step for the input tensor *X* entails a $7\times 7$ convolution in the backbone path, then fusion with *X*. Subsequently, DropPath processing is applied to obtain $X_1$. Subsequently, $X_1$ undergoes fusion with itself after a $7\times 7$ convolution. Subsequently, the combined features undergo a sequence of operations, such as DropPath, Permute, and normalization, to derive $X_2$. Subsequently, the input $X_2$ undergoes processing through a $1\times 1$ convolution and GELU activation function to obtain the output $X_3$. Subsequently, the tensor $X_3$ is subjected to a $1\times 1$ convolution and permutation operation before being combined with the input tensor *X*. Following the fusion process, the final DropPath processing is carried out to derive the fusion feature $X_4$.

### Proposed QAHARep-FPN network

**Figure 2:**
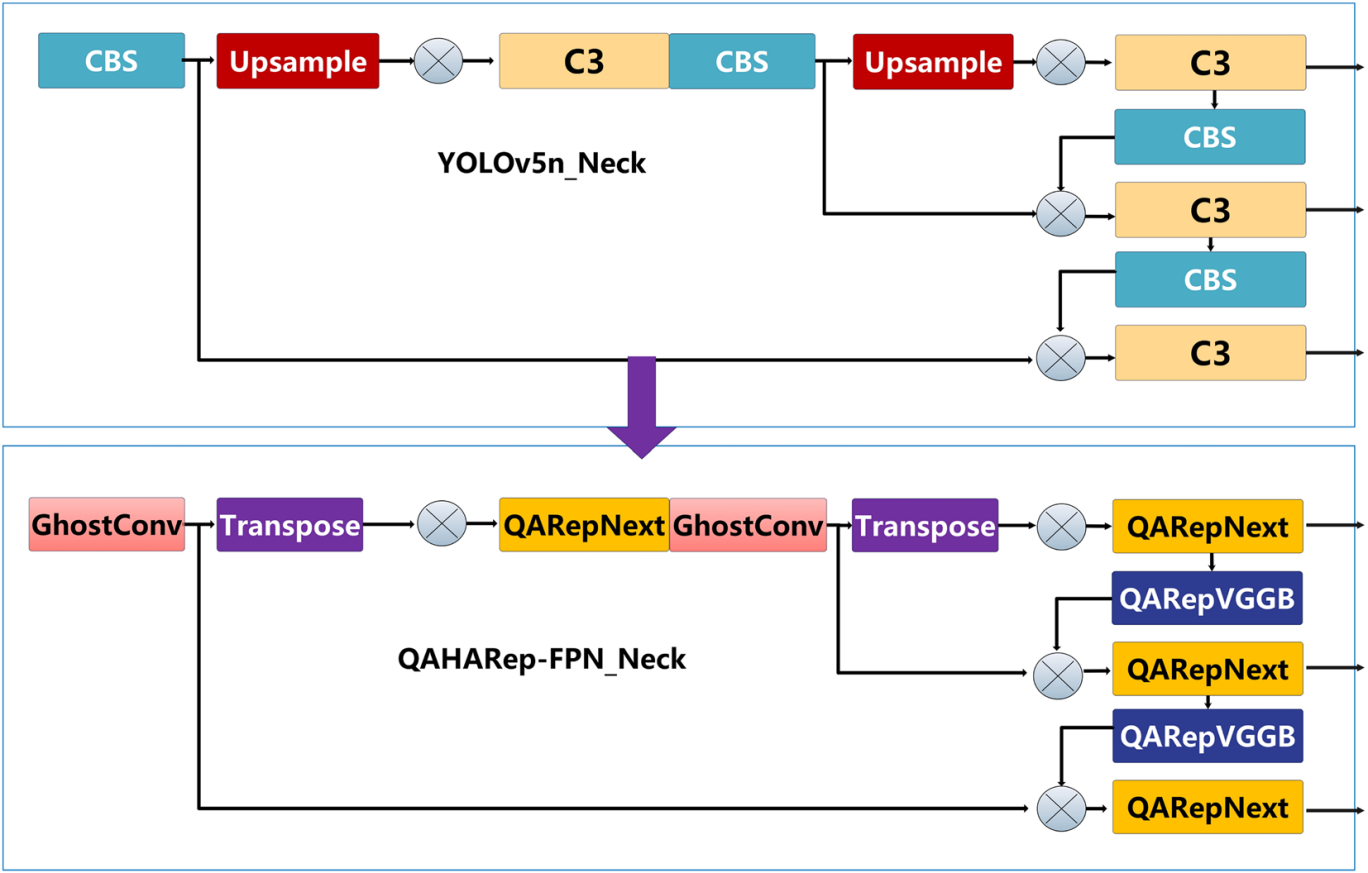
The QAHARep-FPN.

The neck network plays a crucial role in efficiently handling multiscale features from the backbone network. Increasing the quantity of convolutional layers in the neck network might optimize the advantages of fusion. However, it also increases the computational complexity, resulting in an adverse impact on processing efficiency, especially in devices with restricted resources. Network quantization^38,39^ can decrease the cost and computational requirements but may sacrifice detection accuracy. Parameterization^24,25^ can achieve a trade-off between detecting performance and speed, although it may experience a decline in performance when subjected to quantization. In this paper, we integrated network quantization and reparameterization methods to construct a QARepVGG-style^24,25^ feature pyramid network QAHARep-FPN. The QAHARep-FPN structure uses QARepVGGB, QARepNeXt, the Transpose operation^40,41^, and the GhostConv convolution^42,43^. This can be seen in Fig. 2. This approach seeks to achieve an optimal balance between maintaining the accuracy of the detection of fires and achieving fast and efficient inference on devices that have limited resource availability.

**Figure 3:**
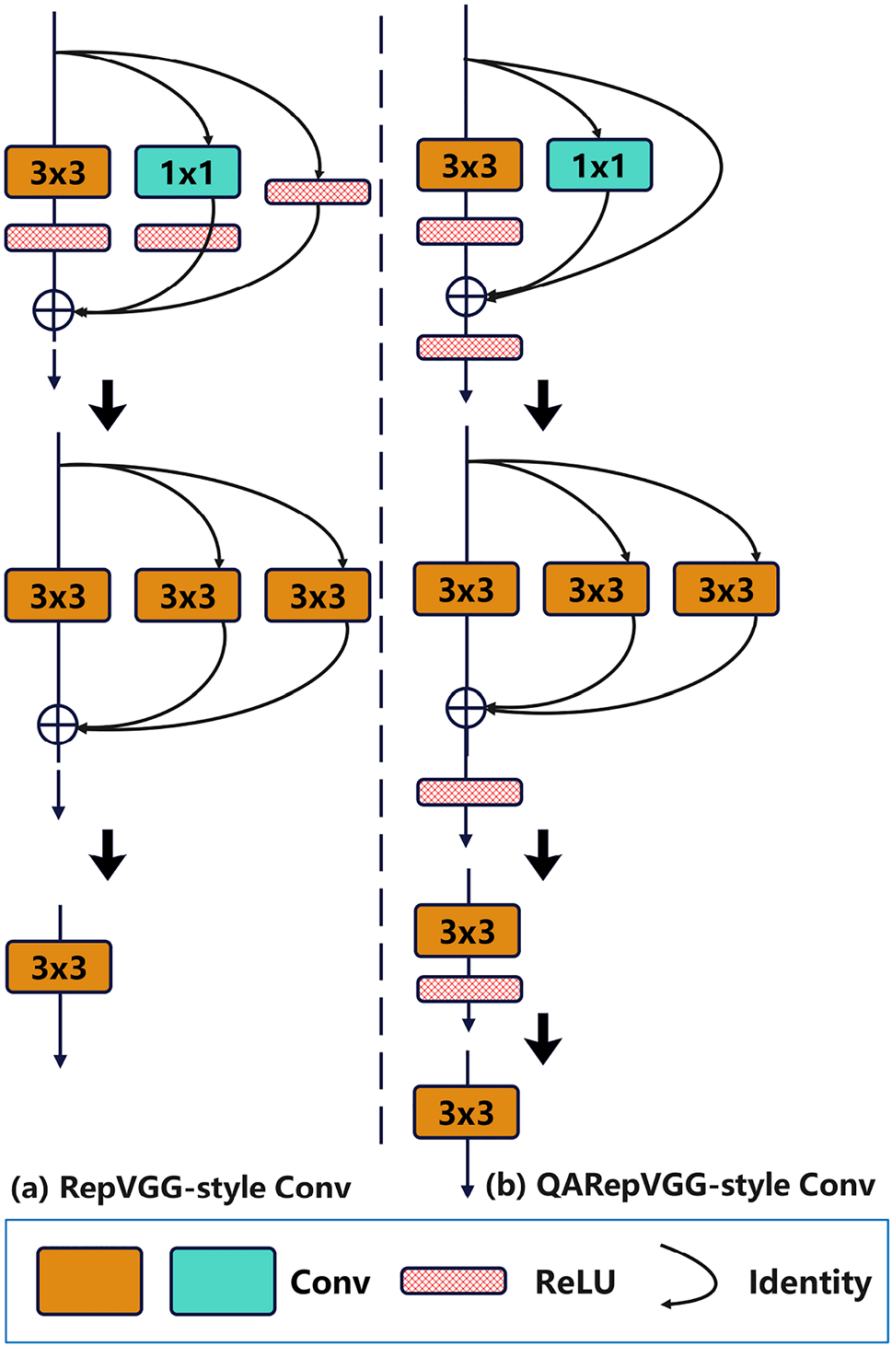
The reparameterization of RepVGG-style Conv and QARepVGG-style Conv.

There are $3\times 3$ convolution, $1\times 1$ convolution, identity, and batch normalization (BN) in both the RepVGG-style and QARepVGG-style convolutional structures (see Fig. 3). During the process of inference, the multi-branch structure is converted into a single-branch $3\times 3$ convolution structure through reparameterization. Nevertheless, the incorporation of three branches results in a covariate shift, which leads to significant performance deterioration during quantization. To address this problem, the QARepVGG-style convolutional structure adds more BN operations and gets rid of BN operations after the $1\times 1$ convolution and identity layers to make the training process more stable. This adjustment greatly enhances the quantization effects of the QARepVGG-style convolutional structure^24,25^. The QARepVGGB module in the paper employed the QARepVGG-style Convolutional structure. We substituted two standard convolutions with the QARepVGGB module.

**Figure 4:**
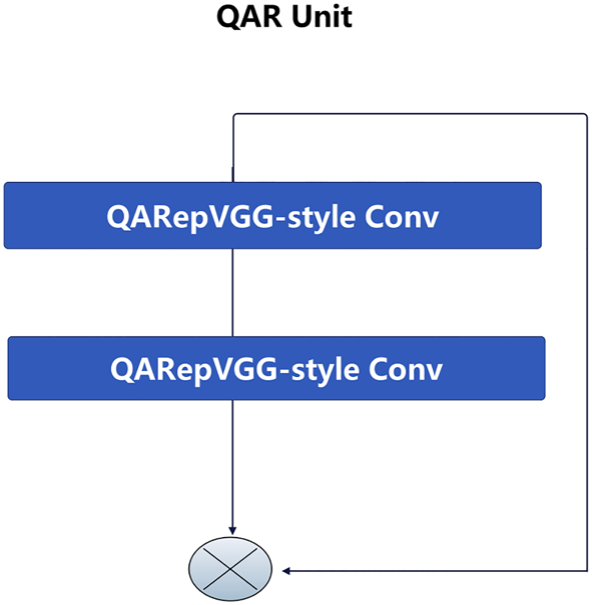
The QAR Unit.

**Figure 5:**
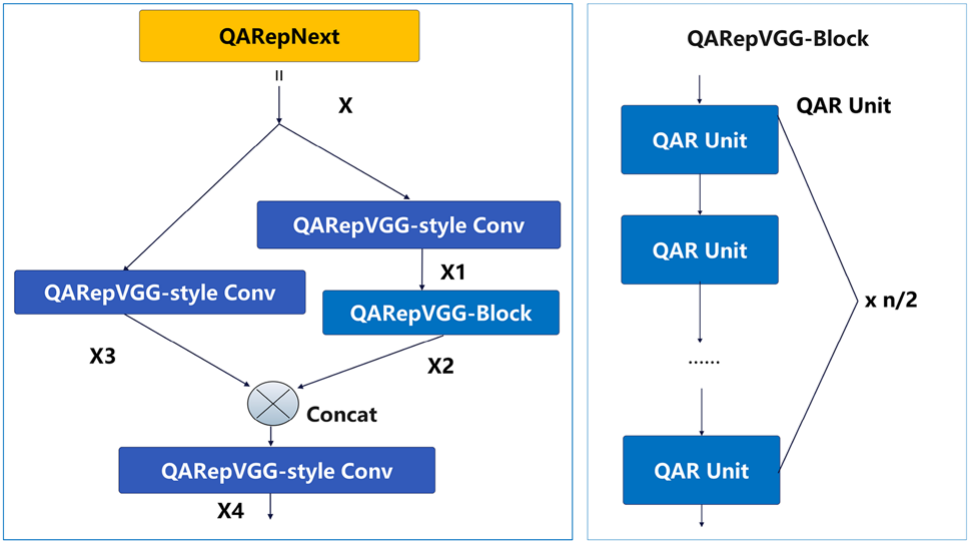
The QARepNeXt and QARepVGG-Block.

Furthermore, we draw inspiration from the EfficientRep network^44^ and design the QAR Unit (Fig. 4) and QARepVGG-Block (Fig. 5). The QAR Unit establishes a linear connection between two QARepVGG-style convolutional structures. The QARepVGG-Block establishes a linear connection between $\frac{n}{2}$ QAR units. The structure of QARepNeXt is illustrated in Fig. 5, using QARepVGGB and QARepVGG-Block. The input variable *X* undergoes the QARepVGG-style convolutional operation in the backbone path, resulting in the generation of $X_1$. Subsequently, $X_1$ is fed into the QARepVGG-Block to undergo more extensive feature extraction, yielding the feature $X_2$. After the QARepVGG-style convolutional operation in the subpath, the feature $X_3$ is combined with $X_2$ through fusion. Ultimately, the combined characteristics undergo the QARepVGG-style convolutional operation to produce $X_4$.

Moreover, we replaced the nn.Upsample operation with the Transpose operation^40,41^. The nn.Upsample mainly uses interpolation to resize images. Although it has some utility in some image-processing applications, it usually requires an immense computational load. Conversely, the Transpose operation can restructure the dimensions of the input tensor to fit different network architectures and task needs. It can also reduce the increased computational load and memory utilization. Transpose operation has notable benefits in terms of improving model adaptability and coping with resource-constrained scenarios. Finally, we used GhostConv^42,43^ to replace two standard convolutions. GhostConv’s channel grouping strategy improves the model’s ability to capture nonlinear features, which improves object detection accuracy and minimizes computing costs.

### Proposed NADH decoupled head

**Figure 6:**
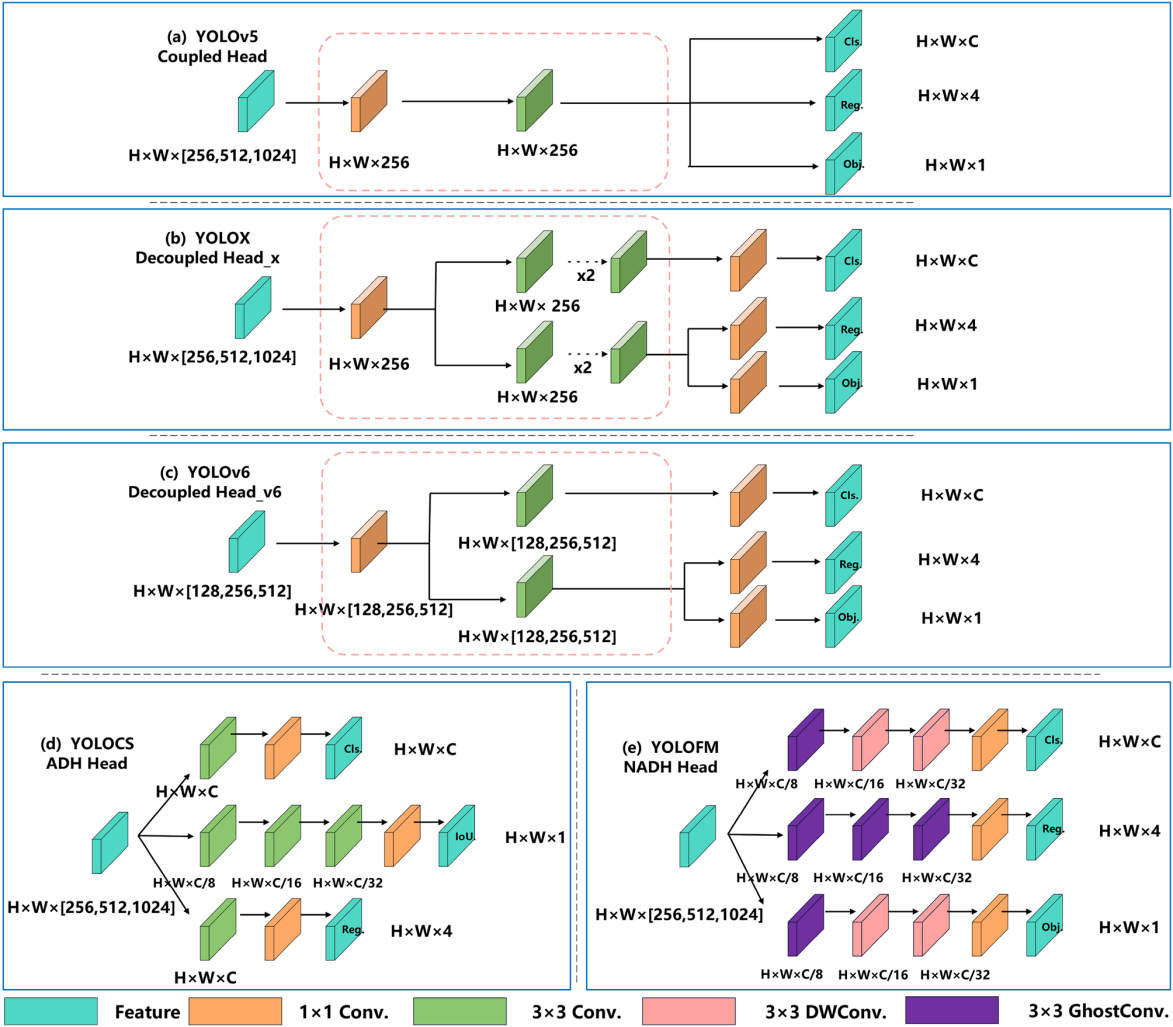
The head network structure comparison between YOLOFM, YOLOCS, YOLOv6, YOLOv5, and YOLOX.

By dealing with the data that the neck network has processed, the head network makes final predictions. The YOLOv5 head network adopts an integration and sharing method for classification and regression tasks (Fig. 6a). This structure potentially results in detecting conflicts for both classification and regression tasks, ultimately leading to subpar performance. YOLOX^8,21^ divides the classification and regression tasks into separate subnetworks. This lets it do more calculations and have more parameters (Fig. 6b). This effectively resolves conflicts but also makes the parameters and computations bigger. YOLOv6^16^ employs hybrid channels as a solution, resulting in parameter reduction at the expense of accuracy (Fig. 6c). YOLOCS^27^ uses asymmetric multichannel compression and decoupling head technology to create separate subnetworks for different detection tasks. This makes the model much more accurate at finding things. However, it has challenges in adjusting the number of convolutional layers and resolving the problem of the vanishing gradient issue (Fig. 6d).

We proposed a new asymmetric multistage channel compression decoupled head named NADH (Fig. 6e). Within NADH, we employed three separate subnetworks to handle classification, object scoring, and bounding box regression. To address the bounding box regression problem, we employed three GhostConv convolutions, which effectively expand the receptive field and augment the parameter count. We used a $3\times 3$ ChostConv convolution and two $3\times 3$ DWConv convolutions to expand the network path for the object scoring and object classification tasks, respectively. At the same time, we compressed the features of the three channels with the same dimension. This allows the three channels to maintain the three-layer convolutional network architecture (Fig. 6).

### Proposed Focal-SIoU loss

The loss function is divided into three parts: classification loss, object scoring loss, and bounding box regression loss. The classification loss evaluates the model’s accuracy in categorizing each bounding box as a member of the corresponding class. The categorical cross-entropy loss is commonly employed to quantify the difference between the model’s classification prediction and the actual label. The calculation procedures for the classification loss are represented by Eqs. (1)–(2).The *N* denotes the total number of classes, $x_i$ represents the predicted value of the current class, $y_i$ represents the probability that the current class will occur given the processing of the activation function, and $y_{i}^{*}$ represents the true value of the current class (which can be either 0 or 1).

1 $$\begin{aligned} y_i= & {} Sigmoid(x_i )=1/(1+e^{-x_i}) \end{aligned}$$

2 $$\begin{aligned} L_{class}= & {} \sum _{n=1}^{N} y_{i}^{*}\log {(y_{i})} +(1-y_{i}^{*})\log {(1-y_{i})} \end{aligned}$$

The object scoring loss quantifies the model’s level of certainty for each bounding box and assesses whether the bounding box encompasses the object. Binary cross-entropy loss is commonly used to quantify the discrepancy between the model’s predicted confidence and the true label. Eq. (3) illustrates the computation process. The $L_{obj}$ denotes the loss of the object score. The $N_{obj}$ denotes the number of positive samples, which corresponds to the number of bounding boxes that include the actual target. The $y_i$ represents the actual label of sample *i*, usually assigned as 1 to indicate the existence of a target or 0 to indicate the lack of a target. The $C_i$ denotes the model’s confidence estimation for sample *i*. This estimate, which uses the sigmoid function, typically falls between 0 and 1.

3 $$\begin{aligned} L_{obj}=- \frac{1}{N_{obj}} \sum _{i=1}^{N_{obj}} \left[ y_{i}\log (C_{i}) +(1-y_{i})\log (1-C_{i})\right] \end{aligned}$$

The bounding box regression loss evaluates the precision of localizing bounding boxes in object detection, which is essential for achieving successful results. The YOLOv5n utilizes the CIoU Loss, which considers the overlap between bounding boxes, the position of the center point, and the difference in size. Nevertheless, the CIoU Loss has difficulties in achieving sample weight balancing, effectively handling overlapping objectives, and adapting to diverse aspect ratios during the training process. We came up with a Focal-SIoU Loss that combines SIoU Loss^29,32^ with Focal L1 Loss to make object detection better. This new loss function considers both positive and negative sample weights, as well as angle, distance, shape, and IoU between the predicted and true bounding boxes. It expedites the convergence of the model and enhances the accuracy in the bounding box regression job (as shown in Eq. (4), where $\gamma$ is usually set to 0.5).

4 $$\begin{aligned} L_{(Focal-SIoU)}=IoU^\gamma L_{SIoU} \end{aligned}$$

**Figure 7:**
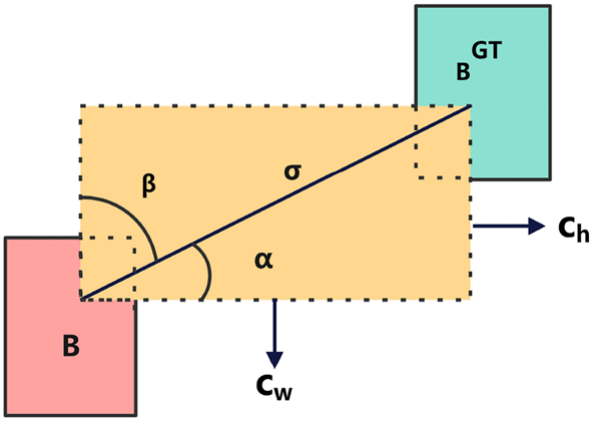
The angle cost.

The calculation procedures for the angle cost are represented by Eqs. (5)–(8). Fig. 7 illustrates that $\Lambda$ is dependent on $\alpha$. $\alpha$ represents the relative angle between the two boxes. The calculation involves utilizing $x=\sin \alpha$ and taking into account the $\frac{\pi }{4}$. When $\alpha$ approaches 0, the angular disparity between the two boxes becomes negligible. When $\Lambda$ approaches 1, this suggests the necessity for optimization of the angle $\alpha$. When $\alpha$ approaches $\frac{\pi }{4}$, and $\Lambda$ is tiny, suggesting that $\beta$ is required to be optimize.

5 $$\begin{aligned} \Lambda= & {} 1-2\sin ^{2}\left( \arcsin (x)-\frac{\pi }{4}\right) \end{aligned}$$

6 $$\begin{aligned} x= & {} \frac{c_{h}}{\sigma } =\sin {\sigma } \end{aligned}$$

7 $$\begin{aligned} \sigma= & {} \sqrt{(b_{c_{x}}^{gt}-b_{c_{x}})^{2} +(b_{c_{y}}^{gt}-b_{c_{y}})^{2}} \end{aligned}$$

8 $$\begin{aligned} c_{h}= & {} \max (b_{c_{y}}^{gt},b_{c_{y} })-\min (b_{c_{y}}^{gt},b_{c_{y} }) \end{aligned}$$

**Figure 8:**
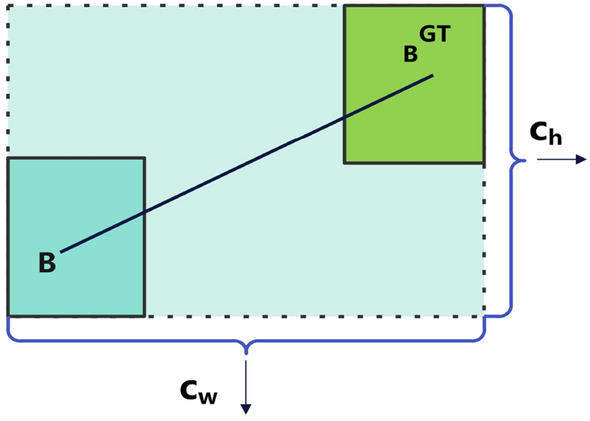
The distance cost.

The calculation procedures for distance cost are represented by Eqs. (9)–(10). Fig. 8 illustrates that $\Delta$ is dependent on $\rho _x$ and $\rho _y$, which quantifies the distance difference between the predicted box and the genuine box. The weight of the distance cost is controlled by $\Delta$, which utilizes $\gamma$ to balance the losses of $\rho _{x}$ and $\rho _{y}$. The $\gamma$ varies in response to changes in $\Lambda$. When $\alpha$ falls and the $\gamma$ increases, the impact of distance cost diminishes, suggesting that distance optimization is hindered. When $\alpha$ approaches $\frac{\pi }{4}$, $\gamma$ decreases, and the importance of distance cost grows, suggesting that distance optimization becomes more prominent.

9 $$\begin{aligned} \Delta= & {} {\sum _{t=x,y}}(1-e^{-\gamma \rho t}) \end{aligned}$$

10 $$\begin{aligned} \rho _{x}= & {} \frac{b_{c_{x}}^{gt}-b_{c_{x} }}{c_{w}},\rho _{y}=\frac{b_{c_{y}}^{gt}-b_{c_{y}}}{c_{h}}, \gamma =2-\Lambda \end{aligned}$$

The calculation procedures for shape cost are represented by Eqs. (11)–(12). When $\theta =1$, the shape cost optimizes the bounding box’s shape and constrains the freedom of the shape. The $\omega _{w}$ and $\omega _{h}$ denote the relative variances in width and height, respectively. Eq. (13) is a representation of the IoU cost calculation procedures. The intersection-over-union ratio (IoU) loss between the predicted and real boxes is measured by the $L_IoUCost$. This quantifies the extent of overlap within the bounding box. The SIoU loss consists of the angle cost, the distance cost, the shape cost, and the IoU cost. Eq. (14) serves as a representation of the calculation procedures.

11 $$\begin{aligned} \Omega= & {} {\sum _{t=w,h}} (1-e^{-\omega t} )^{\theta } \end{aligned}$$

12 $$\begin{aligned} \omega _{w}= & {} \frac{\left| \omega -\omega ^{gt} \right| }{\max ({\omega ,\omega ^{gt} }) },\omega _{h}=\frac{\left| h-h ^{gt} \right| }{\max ({h,h^{gt} }) } \end{aligned}$$

13 $$\begin{aligned} L_{IoUCost}= & {} 1-IoU \end{aligned}$$

14 $$\begin{aligned} L_{box}= & {} 1-IoU+(\Delta +\Omega )/2 \end{aligned}$$

## Experimental setup and data enhancement

The environmental parameters are displayed in Table 1. Batch Size=640×640, Epochs=200, Batch-size=16, Optimizer=SGD, Patience=100, mosaic=1.0, learning rate is 0.01, momentum is 0.937, and weight attenuation coefficient is 0.0005. We used LabelImg software to label fire images and added two types of labels: “fire” and “smoke”. Afterward, we divided the dataset into a 9:1 ratio of training and test sets. We also used image enhancement techniques, including flipping, rotating, and adjusting brightness, to increase the data set. Finally, we acquired a dataset for target detection with 18,644 fire images, which we named FM-VOC Dataset18644. This dataset includes various fire scenarios, such as structure fires, grassland fires, indoor fires, forest fires, road fires, and small target fires. To assess the performance of the model, we employed various metrics such as precision, recall, F1, mean average precision at 50% (mAP50), mean average precision from 50% to 95% (mAP50-95), frames per second (FPS), parameters (Params), and billions of floating-point operations per second (GFLOPs). The calculation procedures for these metrics are shown in Eqs. (15)–(18).

15 $$\begin{aligned} precision= & {} \frac{TP}{TP+FP} \end{aligned}$$

16 $$\begin{aligned} recall= & {} \frac{TP}{TP+FN} \end{aligned}$$

17 $$\begin{aligned} F1= & {} 2\times \frac{precison\times recall}{precision+recall} \end{aligned}$$

18 $$\begin{aligned} mAP= & {} \frac{1}{n} {\sum _{i-1}^{n}} \int _{0}^{1}P(R)dR \end{aligned}$$

**Table 1:** The experimental environment settings.

Schedule	Capacity
Parameters configuration	Windows 10
CPU	Intel (R) Core (TM) i7-8700 CPU @ 3.20 GHz 3.19 GHz
GPU	RTX 2060 (8GB)*1
RAM	16.0 GB
Deployment environment	Python 3.8.10
Deep learning framework	PyTorch 1.11.0
Accelerated computing architecture	CUDA 11.3

## Experimental results and analysis

### The comparative experimental analysis of backbone network improvement

Within Table 2, we conducted a series of integration experiments including the integration of several networks such as InceptionNeXtBlock^45^, FasterNext^46^, ShuffleNetV2Block^47^, BiFormerBlock^48^, CB2D^49^, ELANB^50^, and ConXBv2^51^. The FocalNext network exhibits superior precision and recall compared to the other networks. This illustrates that the FocalNext network can enhance detection precision, while simultaneously minimizing both false positives and false negatives. The FocalNext network exhibits the highest mAP50 and mAP50-95, suggesting superior performance across different IOU thresholds and with a 50% overlap. It possesses the ability to precisely detect fire targets while retaining stability in different situations, which is essential for applications that demand accurate target recognition. The FPS is also relatively high, exhibiting an effective equilibrium between performance and speed. The Parameters and GFLOPs of the FocalNext network are within a modest range, and it performs well in terms of parameter efficiency and computational burden compared to other networks.

**Table 2:** The experimental results of backbone network improvement.

Model	Precision (%)	Recall (%)	mAP50 (%)	mAP50-95 (%)	FPS (%)	Params (MB)	GFLOPs(G)
YOLOv5n	91.8	90.9	95.3	66.8	80.90	6.72	4.1
InceptionNeXtBlock	89.9	89.8	94.2	63.2	75.68	6.71	4.6
FasterNext	90.7	91.5	94.8	64.7	77.21	6.09	3.8
ShuffleNetV2Block	79.0	75.6	82.5	45.2	81.77	3.09	1.5
BiFormerBlock	90.5	91.4	95.1	67.4	61.73	8.05	7.5
CB2D	91.8	90.3	95.5	67.6	63.16	7.07	4.3
ELANB	88.9	88.1	93.6	61.9	76.36	5.29	3.1
ConXBv2	90.1	90.7	94.8	64.8	73.58	6.80	4.2
FocalNext	92.5	92.5	95.8	67.8	77.94	6.64	4.2

### The comparative experimental analysis of neck network improvement

The experimental results shown in Table 3 demonstrate that utilizing the QAHARep-FPN provides substantial benefits in comparison to other combinations of modules or baseline models. QAHARep-FPN achieves superior precision, recall, and mAP50 while still maintaining the computational economy. This illustrates that the QAHARep-FPN framework can attain higher precision in detection. It efficiently minimizes both false positives and false negatives. It exhibits high performance over multiple IoU thresholds and 50% overlap, and it can effectively maintain stability in diverse settings. While the FPS of the QAHARep-FPN network is slightly lower compared to other networks, the disparity is not substantial. It remains feasible to attain an effective equilibrium between performance and inference speed. The QAHARep-FPN network demonstrates superior performance in terms of Params and GFLOPs compared to other networks.

**Table 3:** The experimental results of neck network improvement.

Model	Precision	Recall	mAP50	mAP50-95	FPS	Params (MB)	GFLOPs(G)
Conv+ nn.Upsample+ C3(YOLOv5n)	91.8	90.9	95.3	66.8	80.90	6.72	4.1
AsymptoticFPN	91.0	90.2	94.6	64.1	51.63	5.04	3.4
Conv+ Transpose + QARepNeXt	93.4	90.6	95.8	69.9	50.60	8.64	5.6
SimConv+ Transpose + QARepNeXt	92.8	91.5	95.8	69.7	54.38	8.64	5.6
GhostConv+ Transpose + QARepNeXt	92.6	91.0	95.6	69.6	49.04	8.64	5.6
SimConv + QARepVGGB+ Transpose+ QARepNeXt	91.6	92.0	95.8	68.5	65.05	8.66	5.6
GhostConv + QARepVGGB+ Transpose + QARepNeXt	92.4	92.0	95.8	69.9	57.09	8.63	5.6

### The comparative experimental analysis of head network improvement

The experimental results presented in Table 4 demonstrate the distinct advantages of NADH in enhancing the performance of the YOLOv5n head network, surpassing other head networks. The NADH achieves a precision of 93.8% and a recall of 92.9%, which is significantly better than other head networks. This demonstrates that NADH can attain remarkably high levels of detection accuracy while simultaneously maintaining exceptional recall. The mAP50 and mAP50-95 for NADH are remarkably high, with respectively of 96.2% and 70.6%. This demonstrates that NADH exhibits exceptional performance across various IoU levels.

**Table 4:** The experimental results of head network improvement.

Model	Precision (%)	Recall (%)	mAP50 (%)	mAP50-95 (%)	FPS (%)	Params (MB)	GFLOPs(G)
YOLOv5n	91.8	90.9	95.3	66.8	80.90	6.72	4.1
YOLOX_DH	93.3	92.7	96.2	71.6	39.91	34.20	44.2
YOLOv6_DH	93.0	90.1	95.5	68.0	68.32	7.25	4.6
YOLOCS_ADH	92.6	92.2	95.9	70.3	43.54	22.78	20.3
YOLOFM_NADH	93.8	92.9	96.2	70.6	65.20	13.87	9.3

### The comparative experimental analysis of loss function improvement

The experimental data presented in Table 5 demonstrate that Focal-SIoU outperforms other loss functions to a significant degree. It exhibits high levels of precision and recall, achieving 92.7% and 91.3%, respectively. These results demonstrate that the Focal-SIoU method can achieve accurate object detection with an elevated recall. The Focal-SIoU achieves high mAP50 and mAP50-95 of 95.7% and 68.6% respectively. These results demonstrate that the Focal-SIoU is stable over various IoU overlaps. The Focal-SIoU has a high FPS of 82.29. This demonstrates that Focal-SIoU exhibits a comparatively rapid processing rate in challenges involving high-accuracy object detection. The parameters and GFLOPs are comparable to other loss functions, with both being 6.72 MB and 4.1 G.

**Table 5:** The experimental results of loss function improvement.

Model	Precision (%)	Recall (%)	mAP50 (%)	mAP50-95 (%)	FPS (%)	Params (MB)	GFLOPs (G)
CIoU	91.8	90.9	95.3	66.8	80.90	6.72	4.1
XIoU	91.7	90.7	95.1	66.8	84.84	6.72	4.1
WIoU	92.1	90.5	95.7	66.8	85.92	6.72	4.1
SIoU	91.5	90.5	95.6	67.0	81.38	6.72	4.1
EIoU	91.2	90.6	95.1	65.8	77.59	6.72	4.1
GIoU	90.7	91.1	95.1	66.7	83.27	6.72	4.1
$\alpha$-IoU	90.2	87.1	93.4	65.1	81.38	6.72	4.1
EfficiCIoU-Loss	90.3	88.2	91.7	66.4	81.02	6.72	4.1
Focal-EIoU	90.5	91.5	94.8	65.9	84.27	6.72	4.1
Focal-GIoU	92.0	92.4	95.6	68.2	82.12	6.72	4.1
Focal-DIoU	91.7	91.6	95.4	67.2	81.84	6.72	4.1
Focal-SIoU	92.7	91.3	95.7	68.6	82.29	6.72	4.1

### The ablation experiment

The data shown in Table 6 demonstrates that each improvement has a substantial impact on enhancing the performance of the YOLOv5n fire detection model. Overall, smoke detection is markedly superior to fire detection. The differences could be attributed to the differing visual attributes of smoke targets in comparison to fire targets, making smoke targets more discernible. Moreover, smoke features are simple, whereas fire features are comparatively intricate, posing a greater challenge for the model to comprehend fire in contrast to smoke features. While there are differences, the accurate detection of either smoke or fires in the fire detection can significantly mitigate the risk of fire.

**Table 6:** The ablation experiments.

Model	Num	Class	Precision	Recall	mAP50	mAP50-95	FPS	Params (MB)	GFLOPs(G)
YOLOv5n	N1	All	91.8	90.9	95.3	66.8	80.90	6.72	4.1
		Fire	89.3	89.6	94.2	62.0			
		Smoke	94.2	92.1	96.4	71.5			
FocalNext	N2	All	92.5	92.5	95.8	67.8	77.94	6.64	4.2
		Fire	90.5	93.9	95.1	64.5			
		Smoke	94.5	91.1	96.5	71.1			
FocalNext+QAHARep-FPN	N3	All	93.3	93.3	96.8	72.3	67.98	8.55	5.7
		Fire	91.6	92.6	95.6	66.3			
		Smoke	94.9	94.0	98.0	78.3			
FocalNext+QAHARep -FPN +NADH	N4	All	94.2	94.2	97.2	73.6	66.58	13.87	6.0
		Fire	92.5	93.4	96.0	67.9			
		Smoke	95.9	94.9	98.3	79.4			
FocalNext+QAHARep -FPN+NADH+Focal-SIoU	N5	All	94.9	94.8	97.5	74.7	66.58	13.87	6.4
		Fire	93.5	94.2	96.3	68.8			
		Smoke	96.2	95.4	98.7	80.5			

**Figure 9:**
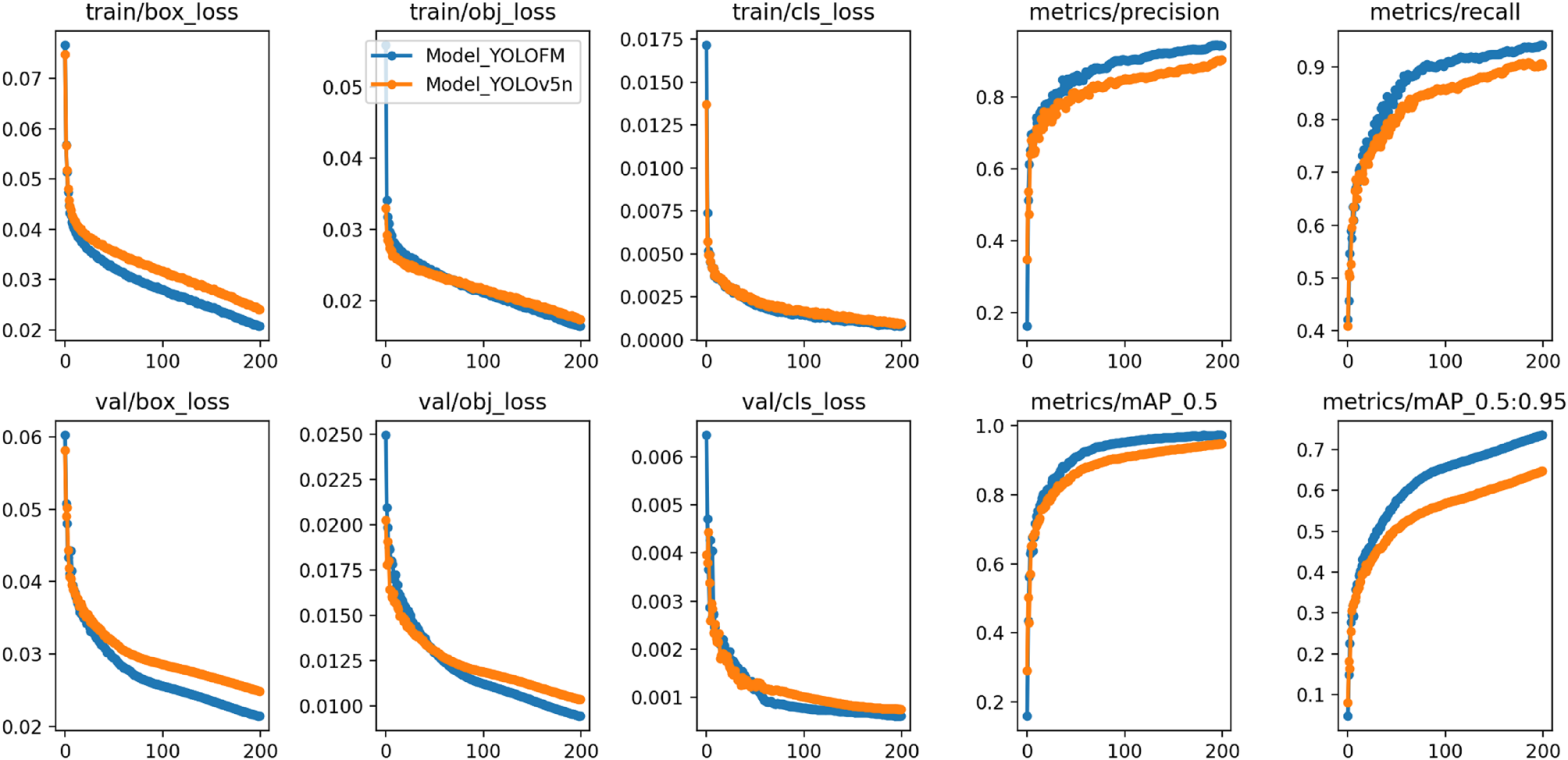
The loss, precision, recall, mAP50, and mAP50-95 training process comparison curves of YOLOv5 and YOLOFM.

**Figure 10:**
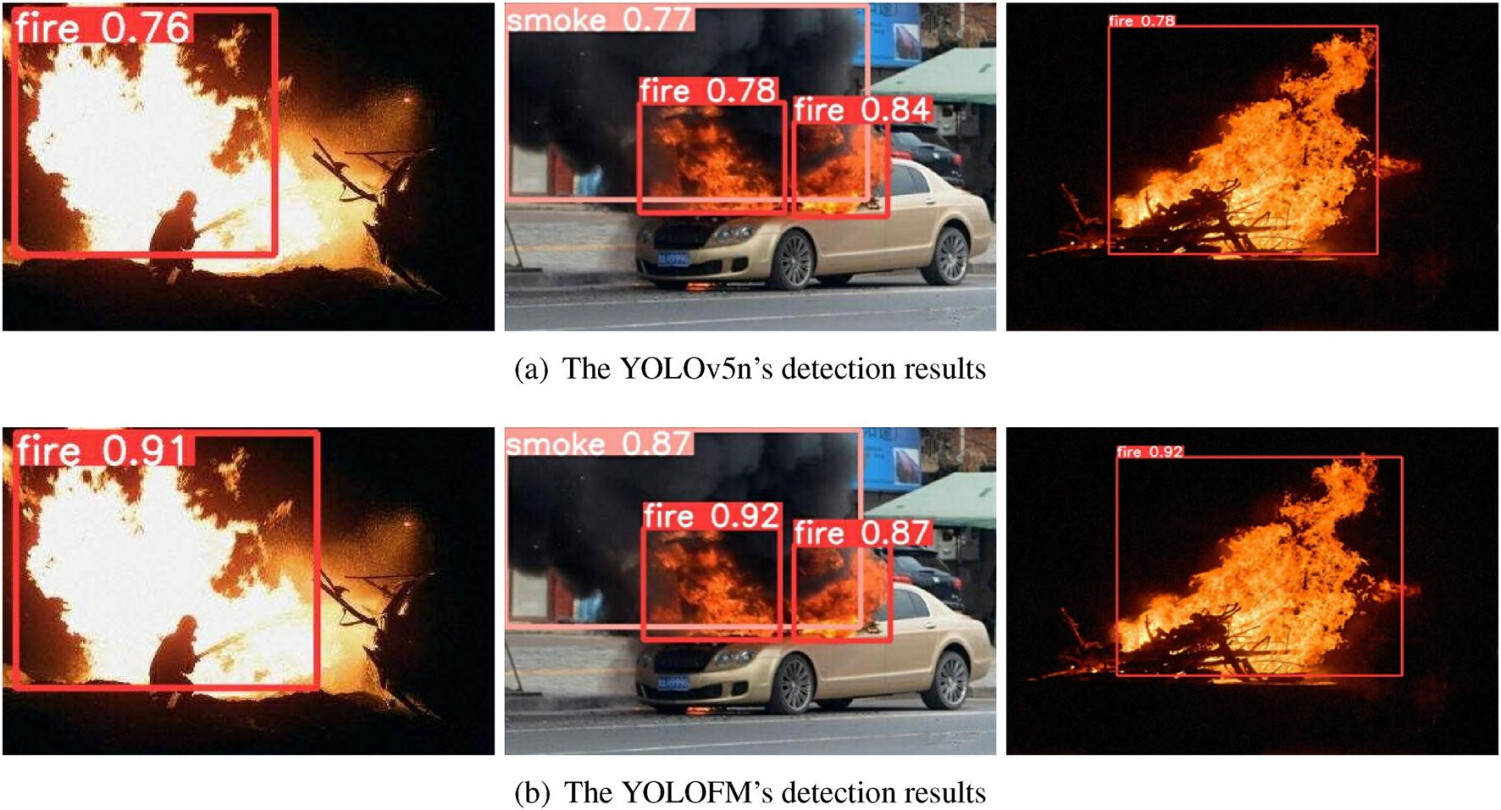
The comparison of real instance detection results between YOLOv5n and YOLOFM.

In Fig. 9, it can be observed that as the number of training rounds reaches 200, the model converges gradually without any signs of overfitting during the training process. The training and verification losses of YOLOFM are lower than those of YOLOv5n, and the downward trend is more pronounced, suggesting a superior capacity to fit the data. As illustrated in Fig. 10, the YOLOFM exhibits greater accuracy in detecting fire and smoke, bettering the YOLOv5n model by a substantial margin. While the FPS of YOLOFM experienced a slight decrease (Table 6), it is apparent that the YOLOFM has achieved notable advancements in improving performance metrics, including precision, recall, mAP50, and mAP50-95. In the context of fire detection tasks, there is a need to balance performance and speed. Generally, greater precision and recall are considered more crucial. When considering the collective impact, the substantial enhancement in overall performance outweighs the minor decrease in FPS, thereby offering a more dependable and precise solution for fire detection assignments. The experimental data from the ablation study offer essential evidence for improving fire detection models, illustrating the effectiveness of these improvements in improving fire detection performance.

### The SOTA comparison experiment

To fully illustrate the originality and effectiveness of the upgraded YOLOFM network,we compared the trained results using the FM-VOC Dataset18644 with other state-of-the-art target recognition techniques, including Fast R-CNN^6^, EfficientDet^7^, SSD^9^, RetinaNet^10^, CenterNet^11^, YOLO series, and EfficientNet-YOLOv3^12^. To ensure fairness, all networks go through the same fine-tuning process during the experiment. The following settings were used: image dimensions of 640x640, 200 epochs, batch size of 16, SGD optimizer, patience value of 100, mosaic factor of 1.0, and learning rate of 0.01. To minimize the impact of software and hardware on model inference time, the experiments are conducted in a controlled experimental setup as shown in Table 1. Table 7 shows that YOLOFM performs well across all parameters, notably in precision, recall, and mAP50. While some algorithms may have slightly better FPS performance in specific conditions, YOLOFM is still an outstanding fire detection algorithm that can properly identify fires. Furthermore, its model parameters and computational complexity are quite low, making it suitable for environments with limited resources. This provides a more dependable and accurate solution for fire detection in equipment with limited resources.

**Table 7:** The SOTA comparison experiment.

Model	Class	Precision (%)	Recall (%)	mAP50 (%)	F1 (%)	FPS (%)	Params (MB)	GFLOPs (G)
SSD^9^	All	86.5	81.9	87.1	84.0	63.70	90.58	60.91
	Fire	82.9	75.5	83.1	79.0			
	Smoke	90.1	88.2	91.1	89.0			
CenterNet^11^	All	96.7	68.7	89.1	80.0	57.98	124.81	69.94
	Fire	96.4	67.5	87.9	79.0			
	Smoke	96.9	69.8	90.3	81.0			
EfficientDet^7^	All	93.0	88.0	92.9	91.0	47.95	14.78	4.63
	Fire	92.2	89.8	92.9	91.0			
	Smoke	93.8	86.2	92.9	90.0			
Fast R-CNN^6^	All	63.8	92.0	89.9	75.0	43.51	136.71	369.74
	Fire	64.6	90.5	88.7	75.0			
	Smoke	62.9	93.4	91.1	75.0			
RetinaNet^10^	All	93.4	90.8	94.5	92.0	43.44	36.35	145.65
	Fire	90.8	88.0	92.2	89.0			
	Smoke	96.0	93.5	96.7	95.0			
YOLOv3^13^	All	89.5	76.6	86.6	83.0	88.86	61.53	65.60
	Fire	86.8	69.0	82.3	77.0			
	Smoke	92.1	84.1	90.8	88.0			
EfficientNet- YOLOv3^12^	All	88.9	69.5	84.0	78.0	50.87	7.22	4.04
	Fire	86.1	59.7	78.1	70.0			
	Smoke	91.7	79.2	89.8	85.0			
YOLOv4^52^	All	92.1	77.5	91.0	84.0	40.96	54.18	59.77
	Fire	89.9	70.6	87.8	79.0			
	Smoke	94.3	84.4	94.1	89.0			
YOLOv4-Tiny^14^	All	90.0	77.9	89.4	84.0	234.70	22.44	6.82
	Fire	88.7	73.0	86.9	80.0			
	Smoke	91.3	82.7	91.8	87.0			
YOLOX (n)^21^	All	86.3	72.1	83.6	78.0	78.41	34.10	55.67
	Fire	84.0	63.6	78.6	72.0			
	Smoke	88.6	80.6	88.5	84.0			
YOLOv7^17^	All	97.1	94.2	97.3	96.0	70.80	37.20	105.13
	Fire	95.8	93.6	96.4	95.0			
	Smoke	98.3	94.7	98.1	96.0			
YOLOv7-Tiny^18^	All	94.8	75.5	93.2	84.0	107.10	6.02	13.19
	Fire	92.0	75.3	90.9	83.0			
	Smoke	97.5	75.7	95.4	85.0			
YOLOv8^19^	All	93.8	94.0	96.8	94.0	71.20	6.91	4.40
	Fire	92.0	93.0	95.7	94.0			
	Smoke	95.5	94.9	97.9	94.0			
YOLOv5n^10^	All	91.8	90.9	95.3	91.0	80.90	6.72	4.10
	Fire	89.3	89.6	94.2	91.0			
	Smoke	94.2	92.1	96.4	91.0			
YOLOFM	All	94.9	94.8	97.5	94.0	66.58	13.87	6.40
	Fire	93.5	94.2	96.3	94.0			
	Smoke	96.2	95.4	98.7	94.0			

## Conclusion

Insufficient feature extraction, excessive network processing complexity, limited deployment on resource-constrained devices, and missed, false, and low accuracy in current fire detection algorithms are discussed in this paper. Optimizing the YOLOv5n algorithm yields the YOLOFM, which is a high-precision, hardware-aware, and quantization-aware fire detection algorithm. The optimization plan includes backbone network rebuilding, neck structure augmentation, asymmetric compression decoupled head introduction, and loss function substitution. These improvements maximize algorithm efficiency and detection performance. However, the complexity of flame features may affect the algorithm’s flame detection performance, resulting in lower performance than smoke detection. This requires attention later.

## Data Availability

Data are available on https://drive.google.com/drive/folders/1BvLKj9jClqHfMbm0o-x6jJJojrfs5tyt?usp=sharing.
